# Supplementary material for: Optimal trade-off between boosted tolerance and growth fitness during adaptive evolution of yeast to ethanol shocks
Source: Biotechnol Biofuels Bioprod. 2024 May 10;17:63. doi: 10.1186/s13068-024-02503-7 (PMC11088041; doi:10.1186/s13068-024-02503-7)

Additional file 2: Appendix S1.

Optimal trade-off between boosted tolerance and growth fitness during adaptive evolution of yeast to ethanol shocks

Ana Paula Jacobus[1,2], Stella Diogo Cavassana[1], Isabelle Inácio de Oliveira[1], Joneclei Alves Barreto[1], Ewerton Rohwedder[3], Jeverson Frazzon[4], Thalita Peixoto Basso[5], Luiz Carlos Basso[3], Jeferson Gross[1] *

1 Bioenergy Research Institute, São Paulo State University, Rio Claro, Brazil; 2 SENAI Innovation Institute for Biotechnology, São Paulo, Brazil; 3 Biological Science Department, University of Sao Paulo, "Luiz de Queiroz" College of Agriculture, University of Sao Paulo, Piracicaba, Brazil; 4 Institute of Food Science and Technology, Federal University of Rio Grande do Sul, Porto Alegre, Brazil; 5 Department of Agri-food Industry, Food and Nutrition, "Luiz de Queiroz" College of Agriculture, University of Sao Paulo, Piracicaba, Brazil.

*jeferson.gross@unesp.br

Appendix S1. Sanger sequencing chromatograms for the wild-type and evolved alleles.

<u>POPULATION 1 (P1)</u>

CYR1 (G4420A) (Ala1474Thr)

PCR: Cyr1P1for/Cyr1P1rev; sequencing: Cyr1P1for

Progenitor
4420 (G)

ATGTTGCCAATCTTTCATATGCAGACTTGCTAAGTGGCGCT

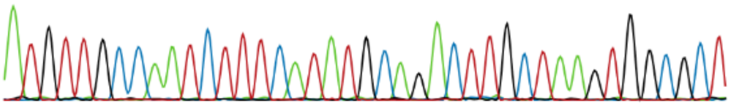

Cycle 20
4420 (A)

ATGTTGCCAATCTTTCATATACAGACTTGCTAAGTGGCGCT

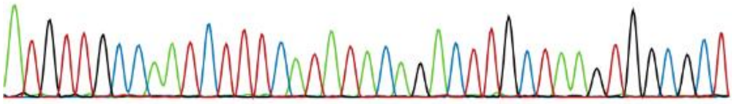

MDS3 (1589InsG) (Val530fs)

PCR: MDS3P1for/MDS3P1rev; sequencing: MDS3P1for

Progenitor
1589 (T)

GTTTCCACCGTATGCCATGGTTCTAGGTAAAGACGCTTTAG

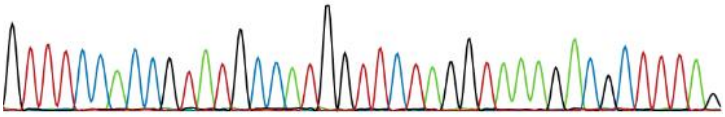

Cycle 20
1589 (G)

GTTTCCACCGTATGCCATGGGTTCTAGGTAAAGACGCTTTAG

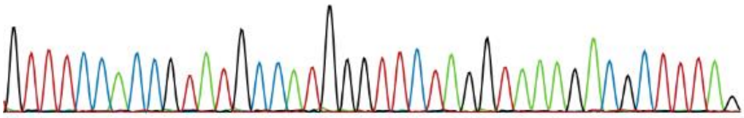

ROM2 (C866T) (Ser289Leu)

PCR: ROM2P1for/ROM2P1rev; sequencing: ROM2P1for

Progenitor
866 (C)

ACATTCTATATCATCTAGATCGATGTCGTTGAACTCATCTA

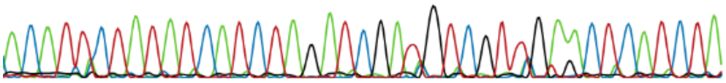

Cycle 20
866 (T)

ACATTCTATATCATCTAGATTGATGTCGTTGAACTCATCTA

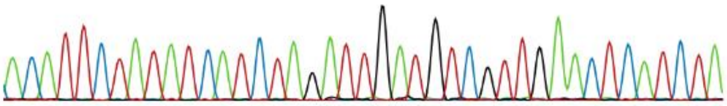

ATH1 (1863DelT) (Phe621fs)

PCR: ATH1P1for/ATH1P1rev; sequencing: ATH1P1for

Progenitor

1863 (T)

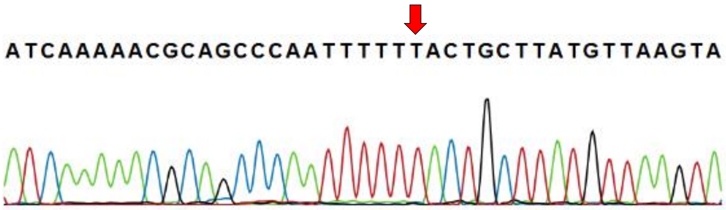

Cycle 40

1863 (A/T)

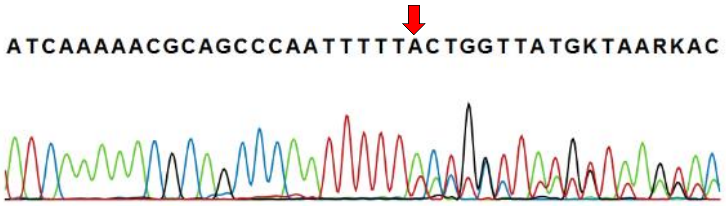

Cycle 60

1863 (A/T)

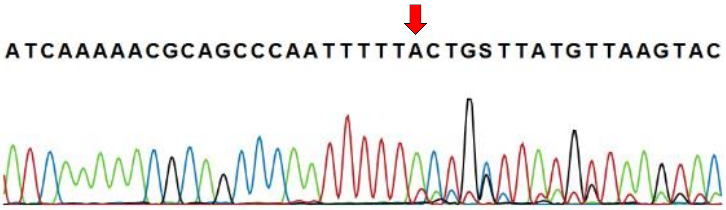

Cycle 68 (end)

1863 (A)

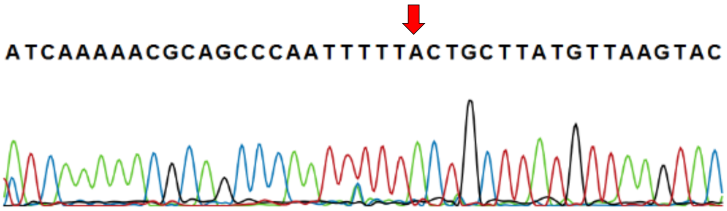

USV1 (C217T) (Gln73Stop)

PCR: USV1for/USV1rev; sequencing: USV1for

Cycle 20

217 (C)

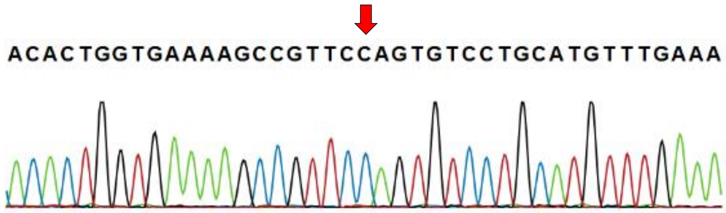

Cycle 40

217 (T)

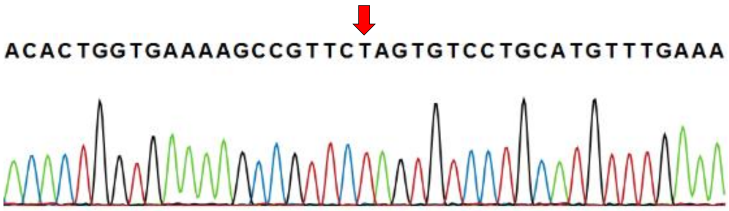

DIG1 (C753A) (Tyr251Stop)

PCR: DIG1P1for/DIG1P1rev; sequencing DIG1P1for

Cycle 60

753 (C)

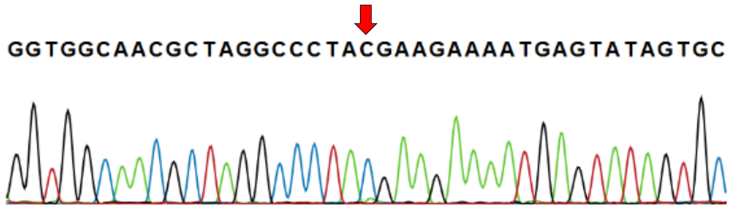

Cycle 68 (end)

753 (A)

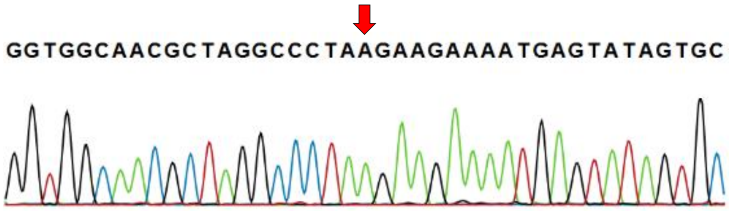

POPULATION 2 (P2)

CYR1 (G2763T) (Leu921Phe)

PCR: CYR1P2for/CYR1P2rev; sequencing: CYR1P2for

Progenitor

2763 (G)

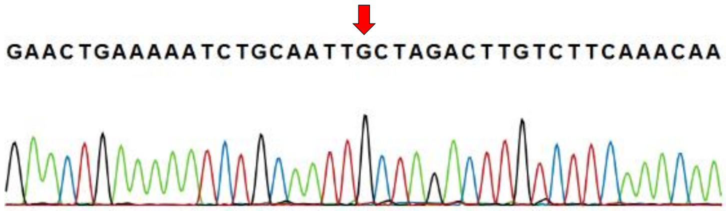

Cycle 20

2763 (T/G)

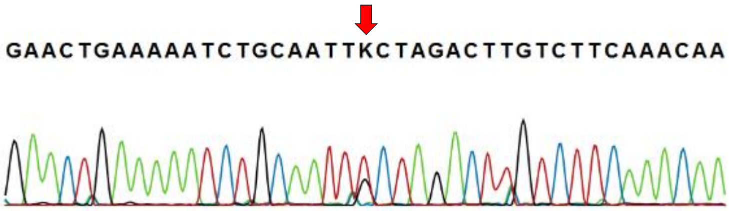

Cycle 40

2763 (T)

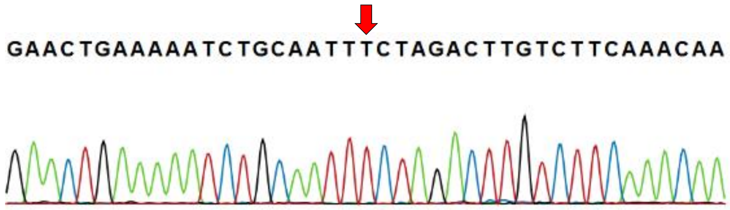

ATH1 (339DelA) (Lys113fs)

PCR: ATHP2for/ATHP2rev; sequencing: ATHP2for

Cycle 20

339 (A)

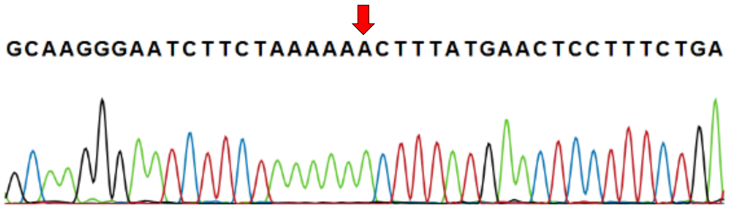

Cycle 40

339 (A/C)

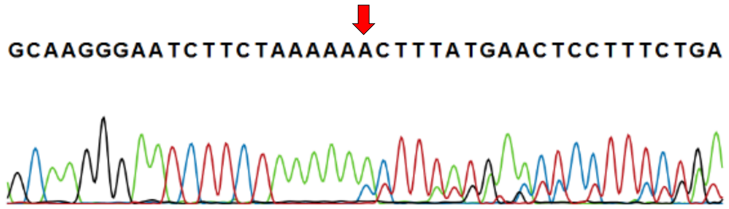

Cycle 60

339 (A/C)

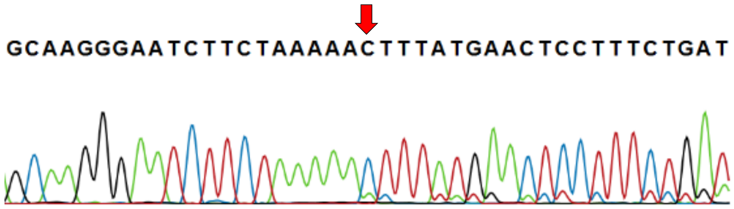

Cycle 71 (end)

339 (C)

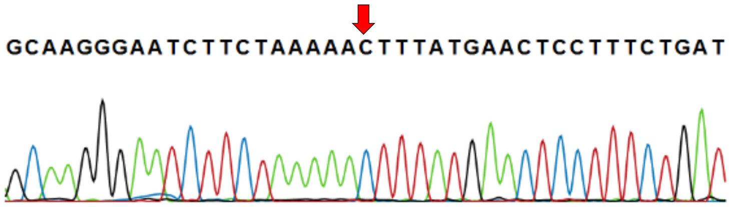

RTT106 (650InsA) (Ile217fs)

PCR: RTT106P2for/RTT106P2rev; sequencing: RTT106P2rev

Cycle 40

650 (T)

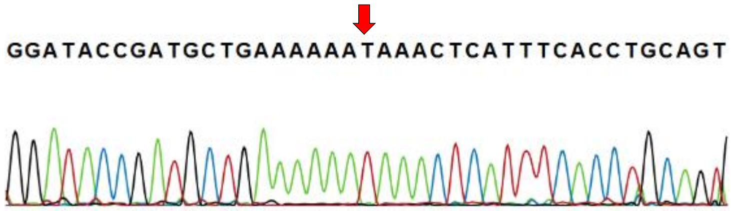

Cycle 60

650 (A)

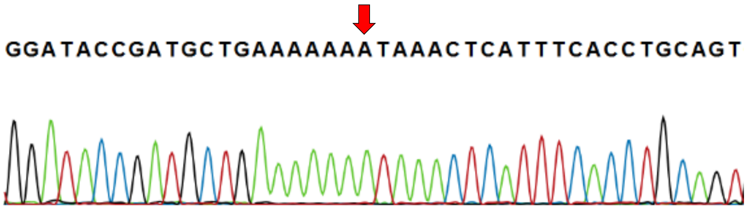

<u>**POPULATION 3 (P3)**</u>

ROM2 (G1318A) (Gly440Arg)

PCR: ROM2P3for/ROM2P3rev; sequencing: ROM2P3for

Progenitor TCGGGGAACATAAGAAGGATGGATTAGTTTACCGTGACGCA

1318 (G)

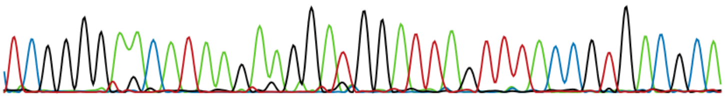

Cycle 20 TCGGGGAACATAAGAAGGATAGATTAGTTTACCGTGACGCA

1318 (A)

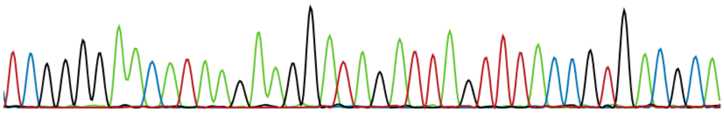

MDS3 (G648T) (Arg216Ser)

PCR: MDS3P3for/MDS3P3rev; sequencing: MDS3P3for

Progenitor GACCCTCAGATAGCGAGAAGGTTCAACCATACCATGCATGT

648 (G)

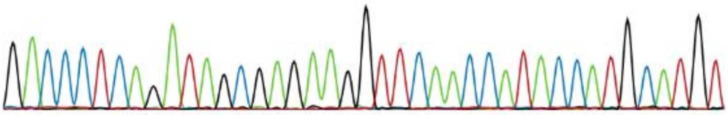

Cycle 20 GACCCTCAGATAGCGAGAAGKTTCAACCATACCATGCATGT

648 (G/T)

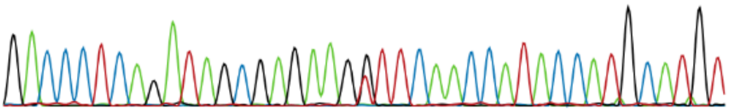

Cycle 40 GACCCTCAGATAGCGAGAAGTTTCAACCATACCATGCATGT

648 (T)

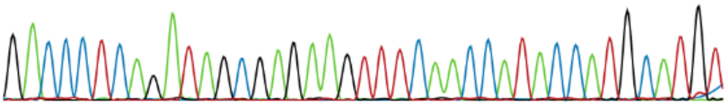

NTH1 (117InsC) (Thr40fs)

PCR: NTH1P3for/NTH1P3rev; sequencing: NTH1P3for

Cycle 20
117 (A)

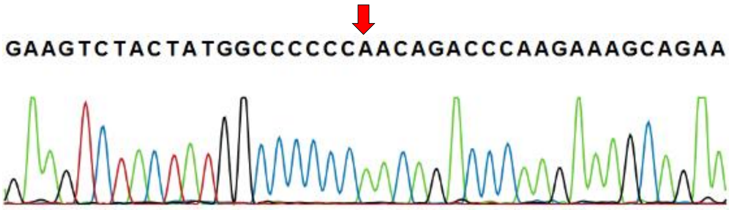

Cycle 40
117 (C)

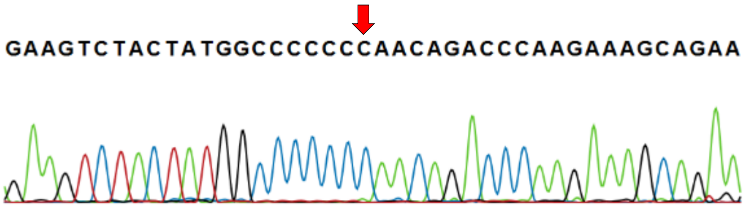

ATH1 (G957A) (Trp319Stop)

PCR: ATH1P3for/ATH1P3rev; sequencing: ATH1P3for

Cycle 20
957 (G)

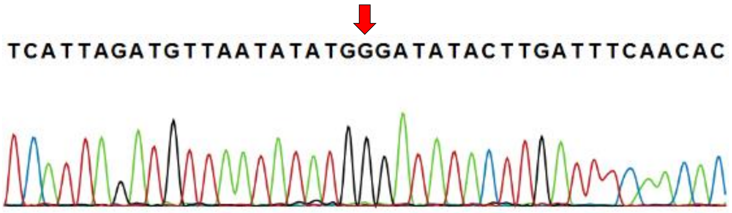

Cycle 40
957 (A)

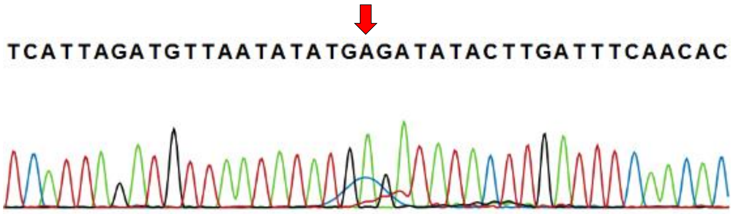

USV1 (G182T) (Arg61Leu)

PCR: USV1for/USV1rev; sequencing: USV1for

Cycle 60
182 (G)

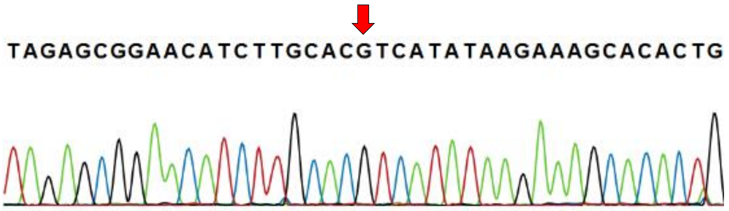

Cycle 80
182 (T)

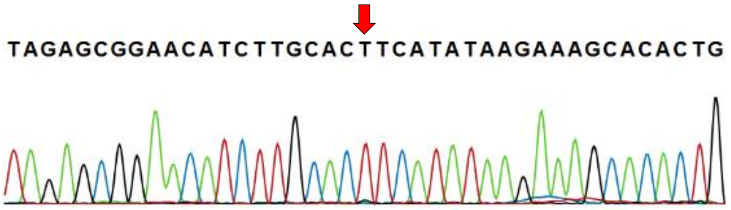

PTR2 (C1436T) (Ser479Leu)

PCR: PTR2for/PTR2rev; sequencing: PTR2rev

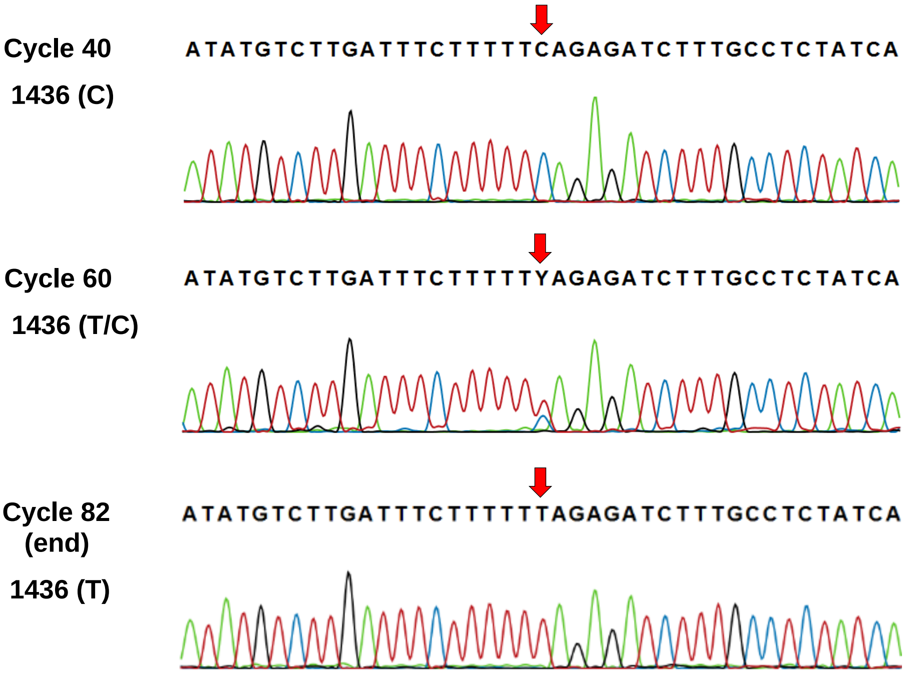

PMD1 (C3039A) (Tyr1013Stop)

PCR: PMD1P3for/PMD1P3rev; sequencing: PMD1P3for

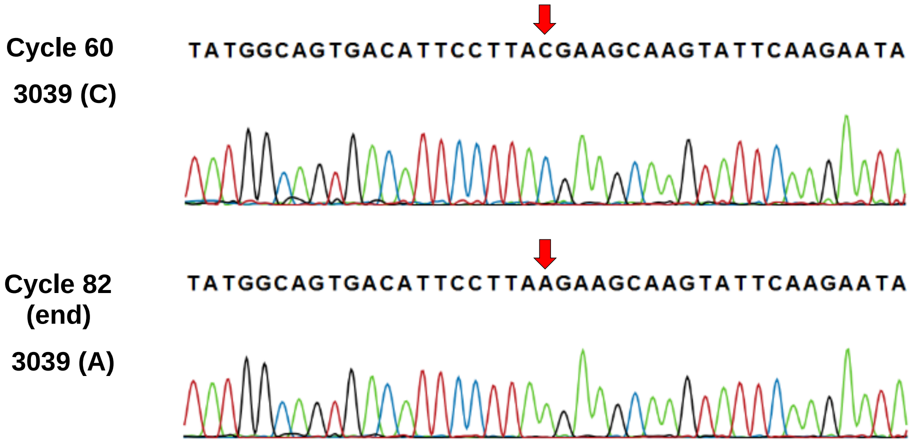

IRA2 (5563DelT) (Cys1855fs), detected in P3c, but not in P3

PCR: IRAP3for/RAP3rev; sequencing: IRAP3for

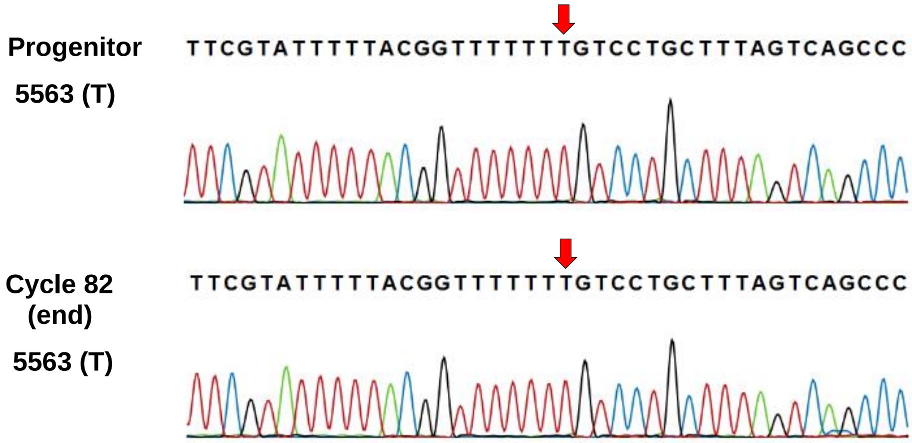

POPULATION 4 (P4)

RAS2 (T2A) (Met1Lys)

PCR: RASP4for/RASP4rev; sequencing: RASP4for

Progenitor AAAAAAAAAATGCCTTTGAACAAGTCGAACATAAGAGAGTA

2 (T)

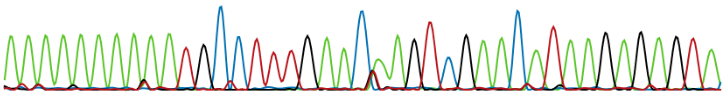

Cycle 20 AAAAAAAAAAAGCCTTTGAACAAGTCGAACATAAGAGAGTA

2 (A)

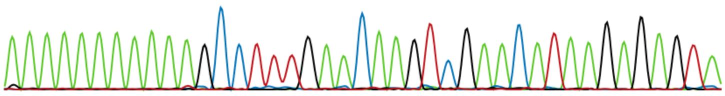

PTR2 (T1084G) (Trp362Gly)

PCR: PTR2for/PTR2rev; sequencing PTR2rev

Progenitor TTATATTCTACCCAATTTATTGGACCCAATACGGTACCATG

1084 (T)

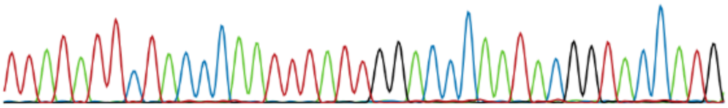

Cycle 20 TTATATTCTACCCAATTTATGGGACCCAATACGGTACCATG

1084 (G)

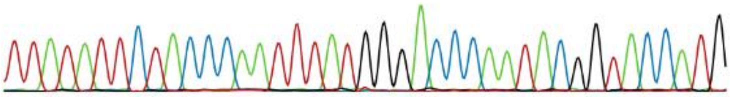

BUD3 (2553DelT) (Phe851fs)

PCR: BUD3P4for/BUD3P4rev; sequencing: BUD3P4for

Progenitor AACAAAGGCAAAGGGTTTTTTGGCGTGTTAAAAAATGTTTT

2253 (T)

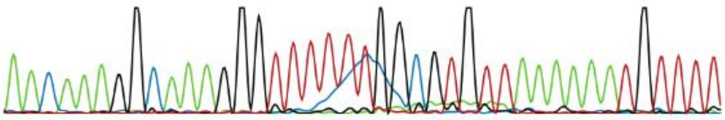

Cycle 20 AACAAAGGCAAAGGGTTTTTTGGCGTGTTAAAAAATGTTTT

2253 (T/G)

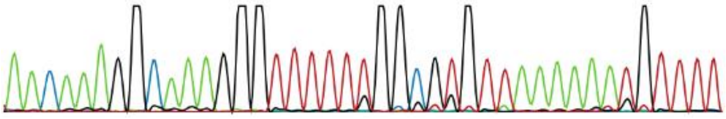

Cycle 40 AACAAAGGCAAAGGGTTTTTGGCGTGTTAAAAAATGTTTTT

2253 (G)

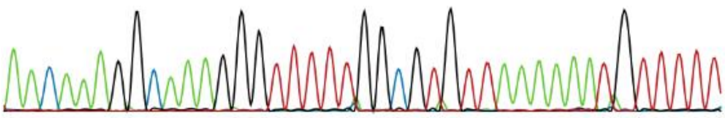

APD1 (3’UTR +2 bp G>T)

PCR: APD1P4for/APD1P4rev; sequencing: APD1P4for

Cycle 40
3’UTR
+2 bp (G)

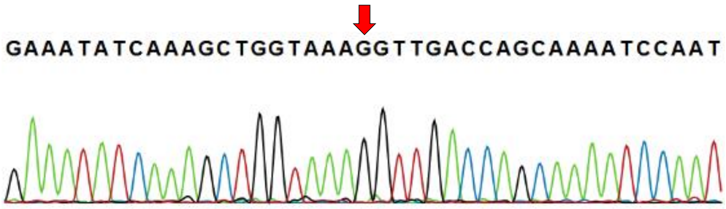

Cycle 60
3’UTR
+2 bp (T)

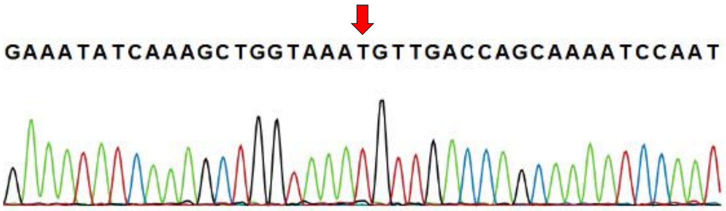

Non-coding DNA between IPL1 and *SRP72* (Inversion: CCTTTGT> ACAAAGG)

PCR: IPL1for/IPL1re; sequencing: IPL1rev

Cycle 40
(CCTTTGT)

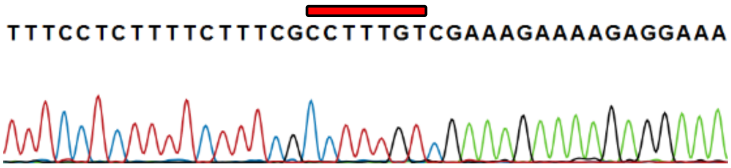

Cycle 60
(ACAAAGG)

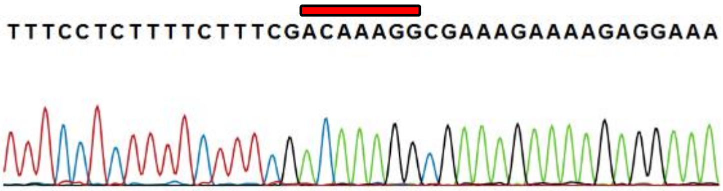

Supplement: Supplementary file 2 — Supplementary Material 2: Appendix S1. Sanger sequencing chromatograms for the wild-type and evolved alleles. Sanger sequencing allowed identification of wild-type and evolved alleles for each population (P1–P4), according to the ethanol shock/recovery cycles. Alleles are indicated by red arrows. Two chromatogram peaks are observed in cycles where the wild-type and evolved alleles coexist in a population. Primers pairs used for PCR amplifications for each sequencing reaction are indicated. [file 13068_2024_2503_MOESM2_ESM.pdf]
