# Supplementary material for: Optimal trade-off between boosted tolerance and growth fitness during adaptive evolution of yeast to ethanol shocks
Source: Biotechnol Biofuels Bioprod. 2024 May 10;17:63. doi: 10.1186/s13068-024-02503-7 (PMC11088041; doi:10.1186/s13068-024-02503-7)
Supplement: Supplementary file 3 — Supplementary Material 3: Fig. S1. Median fluorescence intensities in strains expressing the HSP12-GFP biosensor. Median values of florescence intensities obtained for strains PE-2_H4, P1c, P2c, P3c and cyr1A1474T expressing HSP12-GFP are shown. These data were used to generate the heatmaps in Fig. 3 of the main text. (A) As in Fig. 3B, the time course of GFP fluorescence signal during four consecutive passages (four days) in YPS (without ethanol) for strains is shown. Median florescence values were obtained at the stationary (sta) and logarithmic (log) growth phases. (B) The same data as in (A) expressed as fluorescence fold changes relative to the PE-2_H4 signal at the same time point. (C, D) Time course of GFP fluorescence along 24 h in cells propagating without ethanol (C) and in 8% (v/v) ethanol (D). The data from (C and D) was the basis for the heatmap depicted in Fig. 3C and D of the main text. Statistical analyses refer to the mutant strain being compared to the PE-2_H4 at the same time point. (*) p < 0.05, one way ANOVA followed by Bonferroni post-test for multiple comparisons. Fig. S2. The flocculation phenotype of bud3 disruption depends on the haploid state. Only bud3::MX haploid cells (left) exhibited flocculation. Diploid bud3::MX cells (right) were no longer aggregated. The diploid state was confirmed by PCR of the MAT locus showing the two mating-types. Fig. S3. Ethanol production and cell viability of engineered strains during fermentations. Reverse-engineered strains ath1Δ, cyr1A1474T, and cyr1A1474T/ath1::MX were compared with the parental PE-2_H4 through 14 cycles of sugarcane molasse fermentations. (A) At each new cycle, total reducing sugars concentrations were progressively raised to increase the percentage of ethanol production (v/v). Overall, ethanol production performance of genetically-modified strains was not better than the parental PE-2_H4. (B) Higher ethanol levels decreased the cell viability of tested yeasts. Generally, genetically-m [file 13068_2024_2503_MOESM3_ESM.pdf]

Additional file 3: Figs. S1-S3

Optimal trade-off between boosted tolerance and growth fitness during adaptive evolution of yeast to ethanol shocks

Ana Paula Jacobus[1,2], Stella Diogo Cavassana[1], Isabelle Inácio de Oliveira[1], Joneclei Alves Barreto[1], Ewerton Rohwedder[3], Jeverson Frazzon[4], Thalita Peixoto Basso[5], Luiz Carlos Basso[3], Jeferson Gross[1] *

1 Bioenergy Research Institute, São Paulo State University, Rio Claro, Brazil; 2 SENAI Innovation Institute for Biotechnology, São Paulo, Brazil; 3 Biological Science Department, University of Sao Paulo, "Luiz de Queiroz" College of Agriculture, University of Sao Paulo, Piracicaba, Brazil; 4 Institute of Food Science and Technology, Federal University of Rio Grande do Sul, Porto Alegre, Brazil; 5 Department of Agri-food Industry, Food and Nutrition, "Luiz de Queiroz" College of Agriculture, University of Sao Paulo, Piracicaba, Brazil.

*jeferson.gross@unesp.br

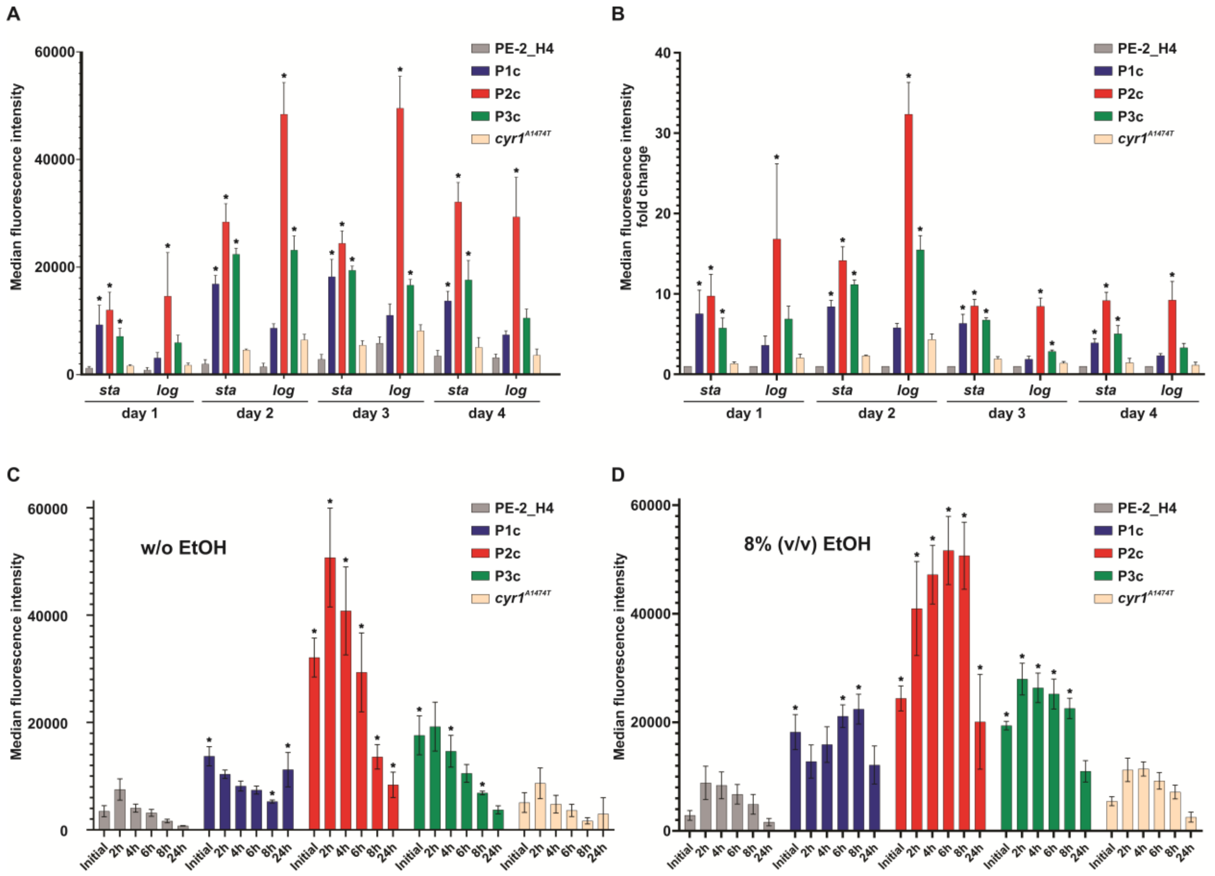

Fig. S1 Median fluorescence intensities in strains expressing the *HSP12-GFP* biosensor. Median values of florescence intensities obtained for strains PE-2_H4, P1c, P2c, P3c and *cyr1*^{A1474T} expressing *HSP12-GFP* are shown. These data were used to generate the heatmaps in Fig. 3 of the main text. (**A**) As in Fig. 3B, the time course of GFP fluorescence signal during four consecutive passages (four days) in YPS (without ethanol) for strains is shown. Median florescence values were obtained at the stationary (*sta*) and logarithmic (*log*) growth phases. (**B**) The same data as in (A) expressed as fluorescence fold changes relative to the PE-2_H4 signal at the same time point. (**C**, **D**) Time course of GFP fluorescence along 24 hrs in cells propagating without ethanol (C) and in 8% (v/v) ethanol (D). The data from (C and D) was the basis for the heatmap depicted in Fig. 3C and D of the main text. Statistical analyses refer to the mutant strain being compared to the PE-2_H4 at the same time point. (*) $p<0.05$, one way ANOVA followed by Bonferroni post-test for multiple comparisons.

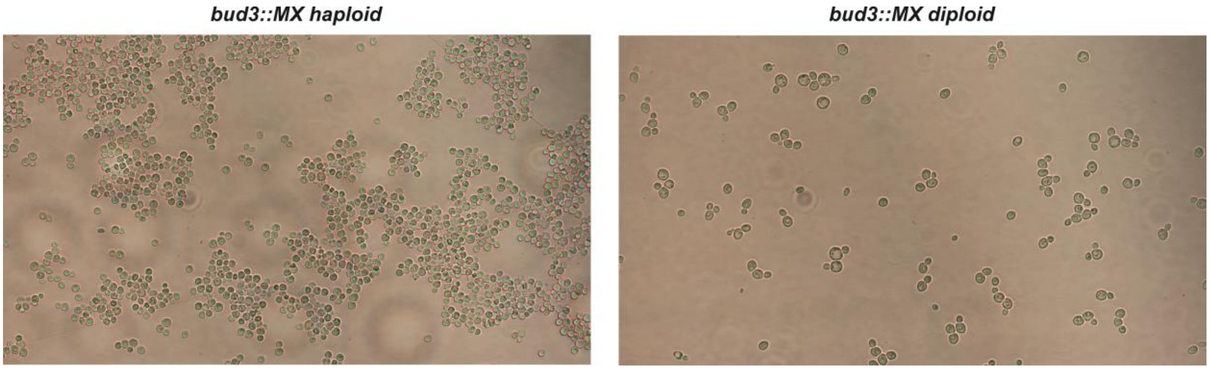

Fig. S2 The flocculation phenotype of *bud3* disruption depends on the haploid state. Only *bud3::MX* haploid cells (left) exhibited flocculation. Diploid *bud3::MX* cells (right) were no longer aggregated. The diploid state was confirmed by PCR of the *MAT* locus showing the two mating-types.

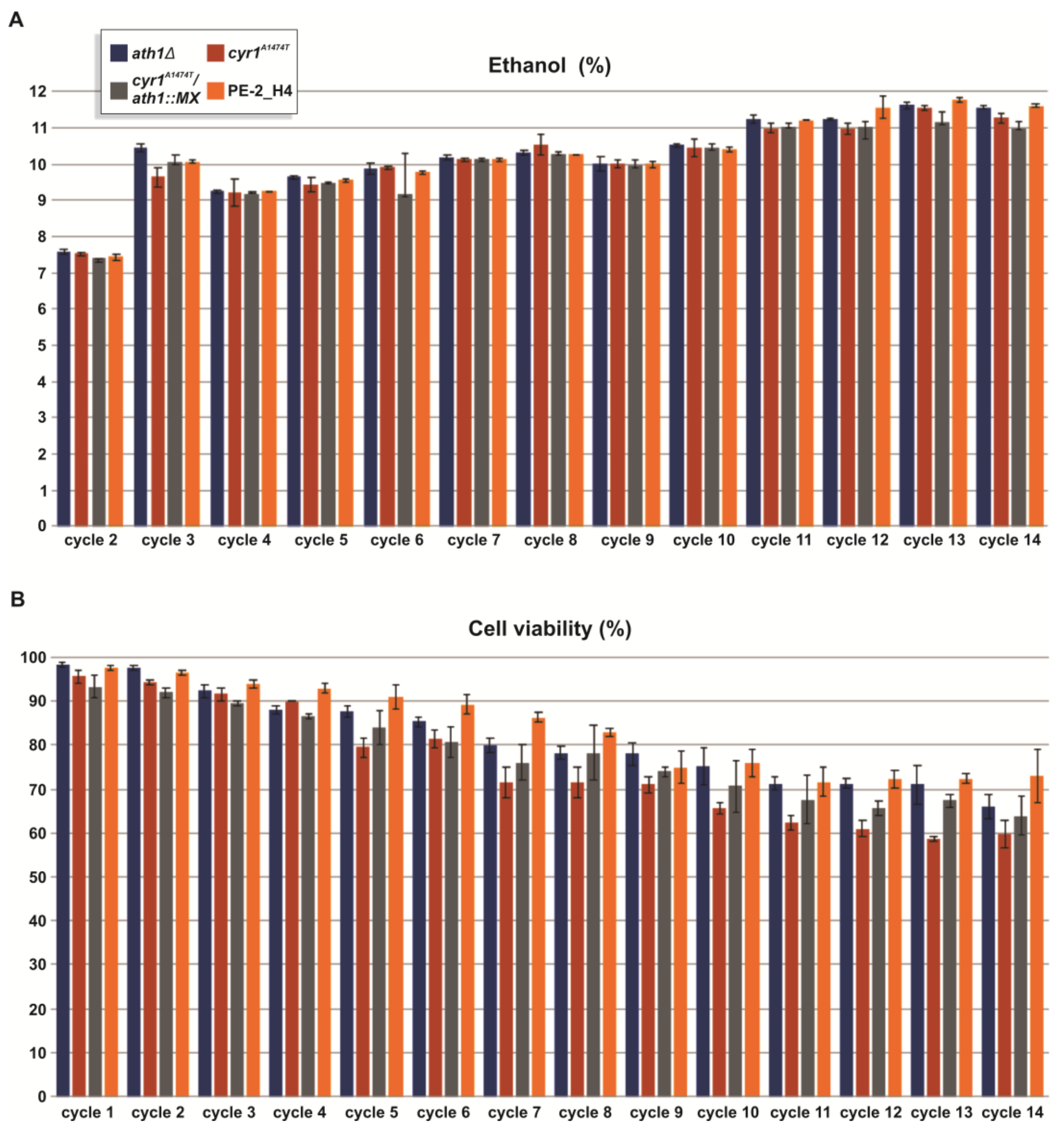

Fig. S3 Ethanol production and cell viability of engineered strains during fermentations. Reverse-engineered strains *ath1Δ*, *cyr1*^{A1474T}, and *cyr1*^{A1474T}/*ath1::MX* were compared with the parental PE-2_H4 through 14 cycles of sugarcane molasse fermentations. (**A**) At each new cycle, total reducing sugars concentrations were progressively raised to increase the percentage of ethanol production (v/v). Overall, ethanol production performance of genetically-modified strains was not better than the parental PE-2_H4. (**B**) Higher ethanol levels decreased the cell viability of tested yeasts. Generally, genetically-modified strains were more sensitive to the ethanol levels than the parental PE-2_H4.
